# Influence of Protein Carbonylation on Human Adipose Tissue Dysfunction in Obesity and Insulin Resistance

**DOI:** 10.3390/biomedicines10123032

**Published:** 2022-11-24

**Authors:** M. Carmen Navarro-Ruiz, M. Carmen Soler-Vázquez, Alberto Díaz-Ruiz, Juan R. Peinado, Andrea Nieto Calonge, Julia Sánchez-Ceinos, Carmen Tercero-Alcázar, Jaime López-Alcalá, Oriol A. Rangel-Zuñiga, Antonio Membrives, José López-Miranda, María M. Malagón, Rocío Guzmán-Ruiz

**Affiliations:** 1 Department of Cell Biology, Physiology, and Immunology, Instituto Maimónides de Investigación Biomédica de Córdoba (IMIBIC), Reina Sofia University Hospital, University of Córdoba, 14014 Córdoba, Spain; 2 CIBER Fisiopatología de la Obesidad y Nutrición (CIBERobn), Instituto de Salud Carlos III, 28029 Madrid, Spain; 3 Nutritional Interventions Group, Precision Nutrition and Aging, Madrid Institute for Advanced Studies—IMDEA Food, CEI UAM+CSIC, 28049 Madrid, Spain; 4 Oxidative Stress and Neurodegeneration Group, Regional Center for Biomedical Research, Department of Medical Sciences, Ciudad Real Medical School, University of Castilla-La Mancha, 13001 Ciudad Real, Spain; 5 Lipids and Atherosclerosis Unit, IMIBIC, Reina Sofia University Hospital, University of Córdoba, 14004 Córdoba, Spain; 6 General and Digestive Surgery Clinical Management Unit, Obesity Section, IMIBIC, Reina Sofía University Hospital, 14004 Córdoba, Spain

**Keywords:** obesity, adipose tissue, insulin resistance, carbonylated protein, redox

## Abstract

Background: Obesity is characterized by adipose tissue dysregulation and predisposes individuals to insulin resistance and type 2 diabetes. At the molecular level, adipocyte dysfunction has been linked to obesity-triggered oxidative stress and protein carbonylation, considering protein carbonylation as a link between oxidative stress and metabolic dysfunction. The identification of specific carbonylated proteins in adipose tissue could provide novel biomarkers of oxidative damage related to metabolic status (i.e prediabetes). Thus, we aimed at characterizing the subcutaneous and omental human adipose tissue carbonylome in obesity-associated insulin resistance. Methods: 2D-PAGE was used to identify carbonylated proteins, and clinical correlations studies and molecular biology approaches including intracellular trafficking, reactive oxygen species assay, and iron content were performed using in vitro models of insulin resistance. Results: The carbonylome of human adipose tissue included common (serotransferrin, vimentin, actin, and annexin A2) and depot-specific (carbonic anhydrase and α-crystallin B in the subcutaneous depot; and α-1-antitrypsin and tubulin in the omental depot) differences that point out the complexity of oxidative stress at the metabolic level, highlighting changes in carbonylated transferrin expression. Posterior studies using in vitro prediabetic model evidence alteration in transferrin receptor translocation, linked to the prediabetic environment. Finally, ligand-receptor molecular docking studies showed a reduced affinity for carbonylated transferrin binding to its receptor compared to wild-type transferrin, emphasizing the role of transferrin carbonylation in the link between oxidative stress and metabolic dysfunction. Conclusions: The adipose tissue carbonylome contributes to understanding the molecular mechanism driving adipocyte dysfunction and identifies possible adipose tissue carbonylated targets in obesity-associated insulin resistance.

## 1. Introduction

Obesity is characterized by the dysregulation of adipose tissue, due in part to an increase in the production of reactive oxygen species (ROS) triggered by the excessive supply of nutrients, promoting the appearance of oxidative stress [1,2]. An excess of ROS causes oxidative cell damage on lipids, leading to lipid peroxidation or binding to amino acid residues causing protein carbonylation [3]. One marker used to measure oxidative stress is protein carbonylation levels, a post-translational modification that occurs by direct interaction with ROS or indirectly as a result of lipid peroxidation [3,4].

Protein oxidative damage preferentially occurs by direct oxidation of the amino acids proline, threonine, lysine, and arginine, through a metal-catalyzed activation of hydrogen peroxide to a reactive intermediate [3]. Classically, protein carbonylation has been considered an irreversible protein modification in response to oxidative stress and is destined only to induce protein degradation in a nonspecific manner. The chemistry of protein carbonylation is complex due to the different biomolecules that are involved. Protein carbonylation is produced as a result of the addition of reactive carbonyl species such as α,β-unsaturated aldehyde 4-hydroxynonenal (4-HNE) [5]. Several methods for measuring protein carbonylation have been implemented, being the determination of protein-bound carbonyls, using most commonly hydrazine derivate 2,4-dinitrophenylhydrazine (DNPH), that introduces detectable functional groups into the oxidized protein and forms hydrazone derivatives (DNP), thus are detected using antibodies (anti-DNP) [3,4].

To parallel molecular analysis, design strategies to reduce ROS production could contribute to developing novel effective therapies for metabolic disease. However, clinical trials focused on small-molecule anti-oxidant therapy have been disappointing [6]. In contrast, the use of antidiabetic drugs (i.e metformin, insulin) reverses metabolic impairment induced by 4-HNE [7]. In the same way, the use of non-invasive drug delivery (i.e insulin, [8] could reduce medical complications (pain, hyperinsulinemia, lipodystrophy surrounding the injection area, …) ameliorate cellular stress, improve glycemic control, and probably reduce protein carbonylation. Further studies are necessary to validate this hypothesis on non-invasive drug delivery.

In adipose tissue, oxidative stress and protein carbonylation are closely associated with intra-abdominal obesity [2,9,10,11,12]. Additional studies carried out by our group, on isolated human adipocytes [13] and by other groups, on total fat [14] demonstrate that insulin resistance leads to an increment of protein carbonylation levels. Although, it has been proposed that protein carbonylation is a causal link between oxidative stress and metabolic dysfunction, several studies evidence that oxidative stress resulted in extensive oxidation and carbonylation of numerous proteins involved in adipose tissue dysfunction including FABP4 or GLUT4, which likely resulted in the loss of protein activity [9,10,12]. Thus, our hypothesis establishes most of the changes observed in adipose tissue that drive insulin resistance are mediated via protein carbonylation and posterior degradation. However, to date, the profile of carbonylated proteins (carbonylome) in the adipose tissue of obese individuals with different insulin sensitivity has not been characterized. Thus, the main objective of this work was to identify the carbonylome of subcutaneous (SC) and omental (OM) adipose tissue in obesity and insulin resistance. The analyses of the carbonylated proteins obtained could describe possible markers of adipose tissue impairment associated with oxidative stress status.

## 2. Materials and Methods

### 2.1. Subjects and Sample Collection

Morbidly obese subjects were recruited at the Lipids and Atherosclerosis Unit of the Reina Sofia University Hospital (HURS, Córdoba, Spain). Clinical characteristics were previously described [13,15,16]. Obese subjects were stratified according to the criteria determined by the American Diabetes Association (ADA) [17] [Obese Normoglycemic (Ob-NG): Fasting plasma glucose (FPG) < 100 mg/dL, HbA1c < 5.7%; obese impaired fasting glucose (Ob-IR) = 100-126 mg/dL, HbA1c: 5.7–6.4% and obese Type 2 diabetes (T2D) > 126 mg/dL, HbA1c > 6.4%]. The Ethics and Research Committee of the RSUH approved the experimental design. Participants gave their informed written consent. Paired plasma and adipose tissue samples SC and OM were obtained from subjects and were kept at −80 °C until use.

### 2.2. Protein Extraction

For 2D proteomic studies, adipose tissue samples were homogenized in cold urea/thiourea buffer [7 M urea, 2 M thiourea, 4%CHAPS, 45 mM Tris, pH 8.4, and Complete protease inhibitors (Sigma Aldrich, St. Louis, MO, USA)] and processed as previously detailed [18].

For immunoblotting studies, adipose tissue samples were homogenized in buffer containing 20 mM Tris-HCl (pH 7.4), 150 mM NaCl, 1% Triton-X-100, 1 mM EDTA, and 1 µg/mL protease inhibitor cocktail (Sigma Aldrich, USA). The homogenate was centrifugated at 13,400 rpm and the supernatant containing cytosolic proteins was kept at −80 °C.

### 2.3. Intracellular ROS Production

The intracellular ROS levels were measured as previously described [15]. Briefly, 50 μg SC and OM depots were combined with 25 μM 2,7′- dichlorofluorescein diacetate (DCF-DA) dye (Sigma Aldrich, USA) and incubated at 37 °C for 30 min in darkness. Flex Station 3 (BioNova Scientific, Madrid, Spain) was used to determine the fluorescence at 485/535 wavelength.

### 2.4. Derivatization of Carbonylated Proteins

Carbonyl groups were detected by derivatization with 2,4-dinitrophenylhydrazine (DNPH). To derivatize carbonylated proteins, 20 μg of protein were treated with a DNPH solution (10 mM DNP in 2 N HCl) for 20 min, and the reaction was stopped with neutralization buffer (2 M Tris-HCl, 30% glycerol).

For two-dimensional gel electrophoresis (2D-PAGE), strips were immersed in a DNPH solution (10 mM DNP in 2 N HCl) during 20 min shaking and the reaction was blocked with equilibration buffer (50 mM Tris-HCl, pH 8.8, 6 M urea, 30% glycerol, 2% SDS) for 10 min shaking.

Detection of protein carbonyl levels was determined by immunoblotting using an anti-DNP antibody (Supplementary Table S1), as previously described [13].

### 2.5. 4-Hydroxynenal (4-HNE) Detection

(4-HNE)-modified protein levels were determined in SC and OM samples as previously described [13]. Nitrocellulose membranes were incubated with a solution of 250 mM sodium borohydride in 100 mM MOPS, pH 8.0 for 15 min. Membranes were washed 3 times with water and subsequently 3 times with PBS solution. Detection of 4-HNE-modified protein levels was determined by immunoblotting using an anti-4-HNE antibody (Supplementary Table S1).

### 2.6. Quantitative Immunoblotting

Briefly, 20 μg of protein extraction/sample were loaded onto 10% SDS polyacrylamide electrophoresis gels and transferred to nitrocellulose membranes (Trans-Blot Turbo Transfer System; Bio-Rad Laboratories, Hercules, CA, USA). Primary antibodies were incubated at 4 °C overnight and peroxidase-conjugated specific secondary antibody was incubated for 1 h at RT (Supplementary Table S1). Immunoreaction was visualized using ECL (Bio-Rad Laboratories, USA). Band intensities were evaluated using Fiji software (ImageJ, https://imagej.nih.gov/ij/ accessed on 1 January 2015.). Ponceau staining was employed as a loading control [13,15].

### 2.7. 2D-PAGE and MALDI-TOF-MS Analysis

Isoelectric focusing and 2D-PAGE analysis were carried out as previously detailed [18]. Briefly, 200 μg of protein from SC and OM samples was resuspended in Rehydration Buffer containing 0.8% of 3-10NL IPG Buffer (GE Healthcare, Madrid, Spain) and incubated for 45 min shaking. Subsequently, samples were added to strip-holders and immobilized pH gradient strips (18 cm, pH 3-10 NL) were rehydrated for 15 h in an Ettan IPGPhor 3 System (GE Healthcare, Spain) following a stepwise voltage: 300 V for 3 h, linear gradient to 1000 V for 2 h, linear gradient to 8000 V for 3 h, and 8000 V until total V/h (40,000) was reached. The temperature during isoelectric focusing was always 20 °C and a maximum of 50 μA/strip. To derivatize carbonylated proteins, strips were immersed in a DNPH solution (10 mM DNP in 2 N HCl) during 20 min shaking and in darkness, and the reaction was blocked with equilibration buffer (50 mM Tris-HCl, pH 8.8, 6 M urea, 30% glycerol, 2% SDS) for 10 min mixing. Strips were equilibrated with equilibrated buffer containing 2% dithiothreitol for 15 min, followed by a 15 min wash with equilibration buffer containing 2.5% iodoacetamide. Finally, proteins were separated on 12% Tris-glycine gels using an Ettan Dalt Six (GE Healthcare, Spain) to detect carbonylated proteins by immunoblot. Gels were transferred to nitrocellulose membranes. Anti-DNP primary antibody was incubated overnight at 4 °C, and peroxidase-conjugated rabbit secondary antibody was administered for 1 h (Supplementary Table S1). Immunoreaction and posterior quantification were detailed above.

To identify carbonylated proteins of interest, a different set of gels were stained with 0.1% Coomassie brilliant blue G-250, 10% ammonium sulfate, 2% phosphoric acid, and 20% methanol. Protein spots were subjected to MALDI-TOF-MS analysis as previously described [13,15].

### 2.8. Iron Content and Histology Staining

Total iron content was measured using an iron assay kit (Sigma-Aldrich, USA) according to the manufacturer’s instructions. Adipose tissue (20 mg) was homogenized in 100 μL of iron assay buffer on ice, and centrifuged (13,500 rpm,10 min at 4 °C) to collect soluble material. For iron content, 40 μL of supernatant was incubated with 5 μL of iron reducer (total iron content) for 30 min at 25 °C. Then, 100 μL of the iron probe was added, mixed, and incubated for 60 min at 25 °C. Finally, the absorbance was measured at 593 nm in a spectrophotometer (FlexStation3, Molecular Devices, Sunnyvale, CA, USA).

Additional, fresh adipose tissue depots from obese subjects were fixed in 10% of paraformaldehyde (PFA) and embedded in paraffin as previously described [15]. Perl’s Prussian Blue staining was used for detection of ferric iron in adipose tissue. Slides (5 µm) of adipose tissue were stained with 2% potassium ferrocyanide in 2% HCl for 2 h and counterstained with 1% neutral red to visualize intracellular iron. Images were obtained using a DC500 camera coupled to a DM5000B Leica microscopy. Total area stained (a.u.) was quantified using Fiji software. Analysis of four/six individuals per group was used.

### 2.9. Culture and Treatment of 3T3-L1 Cell Line

3T3-L1 cells [American Type Culture Collection (Manassas, VA, USA)] were cultured on 12-well plates (3 × 10^3^ cells/cm^2^) in Dulbecco’s Modified Eagle Medium (DMEM) supplemented with 10% of newborn calf serum, 4 mM of glutamine, and 1% of antibiotic-antimycotic solution. At 70–80% confluence, cells were differentiated into adipocytes as previously described [13,15].

Differentiated 3T3-L1 cells (day 9 of differentiation) were exposed to a combination of high concentrations of glucose (4.5 g/L) and insulin (100 nM) (HGHI) for 24 h [19].

Another set of 3T3-L1 cells transfected was exposed to serum collected at baseline from incident-T2D or from non-T2D patients of the CORDIOPREV-DIAB cohort, matched by BMI, waist circumference, and biochemical variables. Cells were treated for 24 h with a medium containing 10% inactivated serum of subjects as previously described [20].

Subsequently, cells were collected and processed for 2D-PAGE carbonylation proteome, immunoblotting, and/or confocal analysis.

### 2.10. Cell Transfection

3T3-L1 cells were seeded on coverslips (30,000 cells per 25 mm round coverslip) and differentiated. On day 3 of differentiation, cells were transfected with Lipofectamine 2000 (Invitrogen, Barcelona, Spain) and HA-tagged transferrin receptor (TfR) expression vector as previously described [21]. 48 h post-transfection, the cells were processed for immunostaining.

### 2.11. Confocal Studies of Transferrin Receptor (TfR) Translocation in 3T3-L1 Adipocytes

For the analysis of iron homeostasis, TfR translocation was measured. Transfected 3T3-L1 adipocytes were exposed to serum from non-T2D and T2D patients for 24 h. After that, cells were exposed to 25 mg/dL of Holo-Tf (Merck KGaA, Darmstadt, Germany) for 20 min and processed for the evaluation of TfR translocation by confocal microscopy.

Briefly, 3T3-L1 cells were fixed in 4% PFA and then immunostained against HA (1:200). Nuclei were stained with 40,6-diamidino-2-phenylindole (DAPI). Samples were examined with an LSM 710 Exciter confocal microscope (Carl Zeiss, Oberkochen, Germany). Image analysis was performed using ImageJ v.1.50 software (NIH, Bethesda, MD, USA) for TfR distribution analysis.

### 2.12. Molecular Modeling of Transferrin

The complex TfR 1 and Tf with iron in the N-Lobe (PDB id code:3s9l) used for molecular modeling is registered in the PDB database (Protein Data Bank, New Jersey). Protein structures were visualized using UCSF Chimera 1.10.1. CLC Sequence Viewer v 7.6.1 software was used to generate the alignments of Tf and carbonylated Tf (C-Tf) sequences. The interacting residues of TfR -Tf-iron were evaluated by Ligprot+ 2.2.5 [22] to generate schematic diagrams of protein-protein interactions.

The 3s9l structure was separate and chain A of Tf was isolated (Wt-Tf) and truncated at the amino acid Pro74 to create a carbonylated Tf (C-Tf). The docking server ClusPro 2.0 [23], was used to perform molecular docking simulations between Tf protein (Wt-Tf or C-Tf) and TfR.

### 2.13. Statistical Analysis

Statistical analysis was performed using GraphPad Prism 7 (La Jolla, CA, USA). Data are expressed as mean ± standard error of the mean (SEM). Correlations were analyzed using Pearson’s correlation test. Statistical differences for single comparisons were determined using unpaired Student’s *t*-test for parametric data or Mann–Whitney test for nonparametric data. For multiple comparisons, we employed one-way or two-way ANOVA tests, and Tukey’s post-hoc test for parametric data. Kruskal–Wallis and Dunn’s post-hoc tests were used for nonparametric data. Results were considered statistically significant with a *p* < 0.05.

## 3. Results

### 3.1. Subject’s Characteristics

Morbidly obese subjects were matched by weight, body mass index (BMI), and waist circumference (Table 1). Fasting glucose and HbA1c were significantly higher between groups confirming the ADA subclassification (Ob-NG, Ob-IR, and Ob-T2D). No differences were observed in insulin levels. Triglyceride levels were higher in Ob-T2D, but no significant differences were observed in other lipid parameters.

Additional oxidative stress biomarkers and iron homeostasis were measured. No differences were detected in other stress parameters such as oxidative stress (GSH, GT, GPx), protein carbonyls (PC), lipid peroxidation products (LPO), nor total nitrites (NOx) (Table 1). Noteworthy, significant correlations between ferritin and glucose (r = 0.39, *p* = 0.0041), HbA1c (r = 0.46, *p* = 0.0027), and insulin levels (r = 0.34, *p* = 0.018) (Supplementary Figure S1) were found, denoting an association between iron and glucose homeostasis in obesity-associated insulin resistance.

### 3.2. Redox Status of Adipose Tissue in Obesity-Associated Insulin Resistance

**Figure 1:**
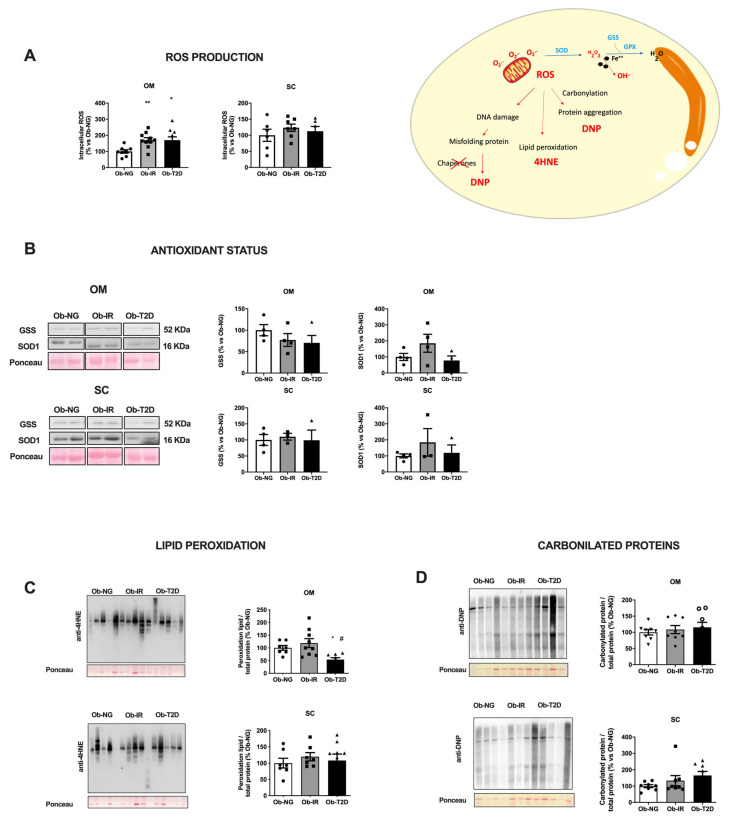
Analysis of the redox status of specific human adipose tissue depots. (**A**) Intracellular ROS in extract from omental (OM) and subcutaneous (SC) human adipose tissue in obese normoglycemic (Ob-NG), insulin resistance (Ob-IR), or diabetic (Ob-T2D) subjects. (**B**) GSS and SOD1 protein expression (immunoreactive bands) in extracts from OM and SC depots; for quantification, protein levels were normalized using Ponceau staining. (**C**,**D**) Quantification of carbonylated proteins using 4-HNE-modified proteins (**C**) and DNP-protein adducts (**D**) in OM and SC human adipose tissue. Immunoblot images (left panel) and quantification (right panel) are shown. Data represent the mean ± SEM (n = 4–6 samples/group). Individual data from different group has been represented by circle (Ob-NG), square (Ob-IR) or triangle (Ob-T2D) * *p* < 0.05; ** *p* < 0.01 vs. Ob-NG; # *p* < 0.05; vs. Ob-IR using One-way ANOVA for parametric data, or Kruskal–Wallis test, for nonparametric data, as posthoc comparisons.

Intracellular ROS production was measured in paired samples of OM and SC adipose tissue from obese-associated insulin resistance subjects. Clear depot-difference in intracellular ROS was observed in adipose tissue from obese-associated insulin resistance subjects (Figure 1A). Thus, the OM depot showed a significant increase in ROS production in obese-associated insulin resistance subjects (Ob-IR and Ob-T2D), while no differences were detected in the SC depot (Figure 1A). No differences were detected in antioxidant status (Figure 1B), elucidating a possible disbalance in antioxidant status in OM fat that could not prevent ROS production.

Additionally, lipid peroxidation (4-HNE) and total carbonylated proteins (DNP) were also measured as adipose tissue stress markers. In general, no differences were detected in these stress markers in adipose tissue from obese subjects (Figure 1C,D). Only, a reduction of 4-HNE content was observed in OM fat, from Ob-T2D (*p* = 0.02 vs. Ob-NG; *p* = 0.01 vs. Ob-IR), probably due to antidiabetic therapies presented in this group, that could reverse 4-HNE content, as previously reported [7] (Figure 1C).

### 3.3. Adipose Tissue Carbonylome in Obesity-Associated Insulin Resistance

**Figure 2:**
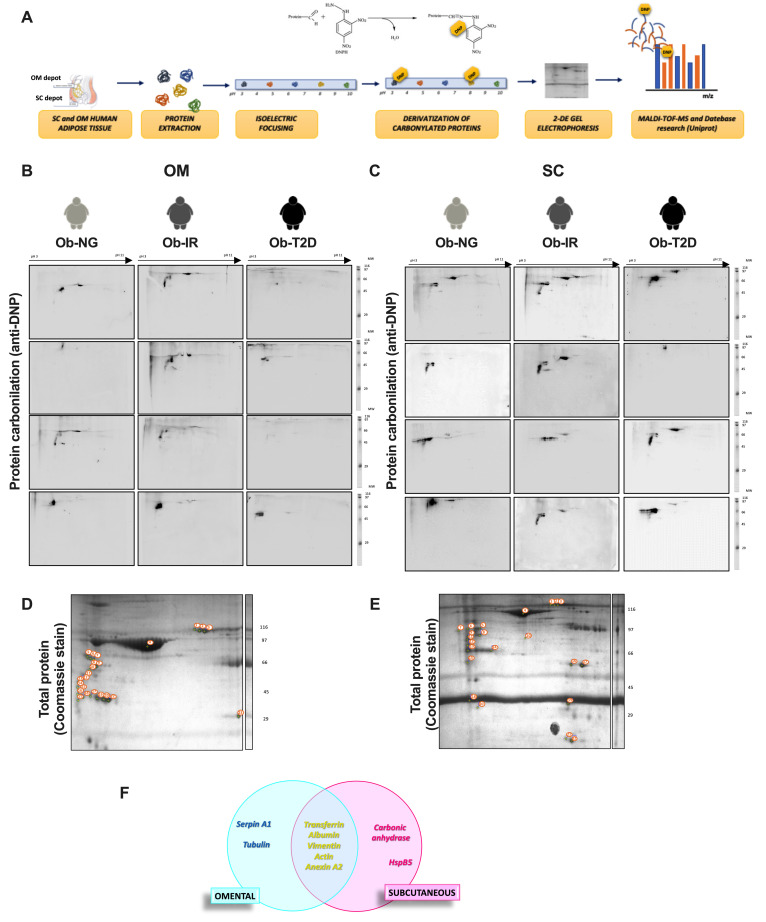
Analysis of the human adipose tissue carbonylome. (**A**) General workflow for the carbonylome analysis in omental (OM) and subcutaneous (SC) human adipose tissue from obese normoglycemic (Ob-NG), insulin resistance (Ob-IR), or diabetic (Ob-T2D) subjects. (**B**,**C**) Two-dimension gel electrophoresis (2D-PAGE) of total carbonylated protein in human adipose tissue in OM (**B**) and SC (**C**) fat depot in obese subjects using anti-DNP antibody. (**D**,**E**) Representative image of total adipose tissue proteome of pooled whole adipose tissue from OM (**D**) and SC (**E**) depot using Coomassie staining. Proteins were separated on a 2-DE gel using 18 cm pH 3–11 NL strips in the first dimension and 10% SDS-PAGE gels in the second dimension. Molecular weight standards were loaded on the second dimension (right). Differentially expressed proteins between the two adipose tissue depots are indicated with arrows. The numbers corresponding to the spot numbers are listed in Table 2. (**F**) Venn diagram showing the number of common and specific carbonylated proteins in (OM) and (SC) human adipose tissue.

Based on previous data obtained in depot-specific differences in intracellular ROS production, a carbonylated protein fingerprint (carbonylome) was analyzed to identify possible changes in the cell-signaling pathways influenced by protein carbonylation implicated in the adipose tissue insulin response. Figure 2A showed the workflow of the 2D-PAGE proteomic approach. Total soluble proteins were separated by isoelectric point (horizontally) and molecular weight (vertically) and the subset of carbonylated proteins in OM (Figure 2B) and SC (Figure 2C) adipose tissue in obese-associated insulin resistance subjects (Ob-NG, Ob-IR, and Ob-T2D) were identified.

A total of 23 spots were detected in the OM depot and 22 spots in the SC depot (Figure 2D,E). Analysis of the proteomic data identified a total of 14 carbonylated proteins expressed in adipose tissue, 7 detected in OM and 7 detected in the SC depot (Table 2). Among them, five proteins including Tf, albumin, vimentin, actin, and annexinA2, were common between fat depots. Additionally, two depot-specific proteins were also detected, carbonic anhydrase and heat shock protein beta-5 in SC fat and, serpin A1 and tubulin in OM fat depot (Figure 2F).

**Figure 3:**
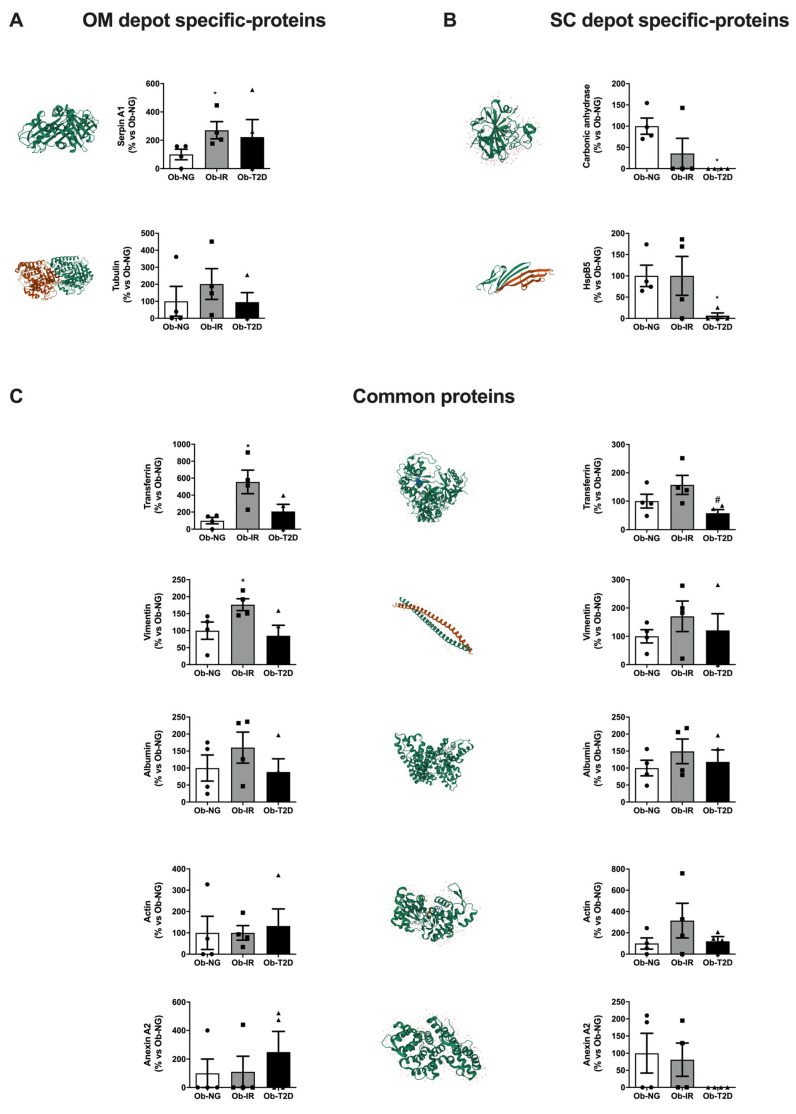
Quantification of carbonylated proteins expression levels identified by 2D-PAGE in omental and subcutaneous adipose tissues. Selected specific omental (OM) (**A**) or subcutaneous (SC) (**B**) adipose tissues and common (**C**) protein spots identified in Table 2 were analyzed using ImageJ software. Tridimensional structure images based on PDB protein (left panel) and quantification (right panel) of carbonylated spots using ImageJ are shown. Data represent the mean ± SEM (n = 4–6 samples/group). Individual data from different group has been represented by circle (Ob-NG), square (Ob-IR) or triangle (Ob-T2D) * *p* < 0.05 vs. Ob-NG; # *p* < 0.05 vs. Ob-IR using Kruskal–Wallis test as post-hoc comparisons.

Specific spots identified corresponding to protein carbonylation expression were quantified and results are shown in Figure 3. In OM fat, only the expression of carbonylated protein serpin A (*p* = 0.013), a serine inhibit protease involved in the control of ER stress response among other functions, and carbonylated transferrin (*p* = 0.036), an iron protein transport and carbonylated vimentin (*p* = 0.028) were significantly higher in Ob-IR vs. Ob-NG (Figure 3A–C). In contrast, only significant changes were detected in ob-T2D subjects in SC fat, showing a significant reduction of carbonylated protein expression of carbonic anhydrase (*p* = 0.028) and HpsB5 (*p* = 0.028) vs. Ob-NG (Figure 3B,C). Data show differences in carbonylated protein profile expression between fat depots, which could compromise ER stress response and glucose homeostasis via iron metabolism and cytoskeletal response, specifically in OM fat.

### 3.4. The Effect of Hyperglycaemia/Hyperinsulinemia on Adipose Tissue Carbonylome

To unravel the molecular mechanism related to insulin resistance and the carbonylation process in adipose tissue, an in vitro model of insulin resistance based on exposure to high glucose and high insulin (HGHI) concentrations was carried out in 3T3-L1 adipocytes (Supplementary Figure S2A). First, Akt phosphorylation was measured in response to insulin stimulus. Data showed a decrease in pAkt/Akt ratio in the HGHI group, validating insulin resistance status in the model (Supplementary Figure S2B). Following, intracellular ROS levels and 4-HNE content were increased in the HGHI group, suggesting an oxidative role of hyperglycemia/hyperinsulinemia on adipocytes (Supplementary Figure S2C,D). In contrast, no differences were observed in DNP adducts (Supplementary Figure S2E) according to human samples observed.

Finally, a similar workflow based on the 2D-PAGE approach (Supplementary Figure S2F) was carried out to identify possible differences in protein carbonylation fingerprints (Supplementary Figure S2G). Only two carbonylated proteins were significantly different between groups: HSPA4, a chaperone protein involved in protein degradation, and VCP, a component of the ternary complex, involved in protein degradation via the proteasome. These data showed that the hyperglycemia/hyperinsulinemia in vitro model promotes protein carbonylation in proteins related to ER stress and protein degradation. Similar results were observed in human samples, showing carbonylation of Heat shock protein β-5, (CRYAB_HUMAN) a chaperone involved in protein degradation via proteasome (Table 2). These data evidence a possible role of insulin resistance on ER stress that could be related to protein carbonylation and dysfunction and lead to protein degradation. However, no positive results related to metabolic proteins were detected evidencing several limitations to performed functional studies on metabolic carbonylated proteins detected in human adipose tissue.

### 3.5. Impact of Iron Homeostasis on Adipose Tissue in Obesity-Associated Insulin Resistance

**Figure 4:**
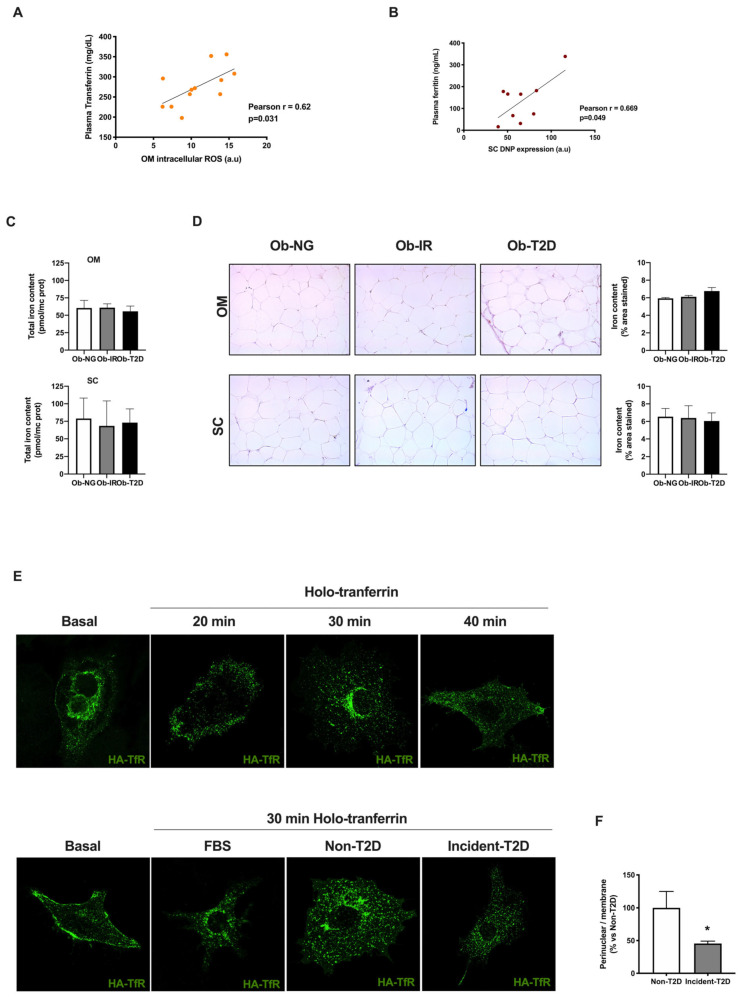
The effect of obesity and insulin resistance on iron homeostasis in human adipose tissue. (**A**) Correlation between plasma transferrin and OM intracellular ROS. (**B**) Correlation between plasma ferritin and SC DNP expression. (**C**) Total iron content in omental (OM) and subcutaneous (SC) human adipose tissue from obese normoglycemic (Ob-NG), insulin resistance (Ob-IR), or diabetic (Ob-T2D) subjects. (**D**) Prussian blue staining (left panel) and image and total area content (right panel) in OM (upper) and SC (lower) human adipose tissue. Representative confocal micrographs of transferrin receptor conjugated with anti-HA (HA-TfR) translocation in 3T3-L1 adipocytes in basal conditions or after holo-transferrin stimulus. (**E**) Representative confocal micrographs of transferrin receptor conjugated with anti-HA (HA-TfR) translocation in 3T3-L1 adipocytes exposed to FBS (control) or sera from non-T2D or incident T2D subjects, in basal conditions or after 30 min of holo-transferrin stimulus. (**F**) Quantification of ratio perinuclear reticular structures/ membrane labeling intensity of confocal images (8–10 micrographs/condition). * *p* < 0.05 vs. non-T2D using unpaired *t*-test.

Positive correlations between iron plasma parameters and adipose tissue stress markers were observed. Thus, intracellular OM ROS production correlates with Tf plasma levels (r = 0.62, *p* = 0.031) (Figure 4A) and total SC DNP content correlates with plasma ferritin levels (r = 0.669, *p* = 0.049) (Figure 4B) suggesting a relationship between adipose tissue stress and alteration in iron metabolism.

First, to analyze possible alteration in iron transport via protein carbonylation of transferrin, we measured iron content in both adipose tissue depots from obese subjects. No differences were observed in total iron content between groups in OM or SC fat depots (Figure 4C). In this line, Perl’s Prussian Blue staining for adipose tissue slides showed similar results in ferric staining in Ob-IR vs. Ob-NG groups (Figure 4D).

Following, to evaluate possible binding alterations between carbonylated transferrin and its receptor, transferrin receptor (TfR), translocation studies were performed. To this end, adipocytes were previously exposed to prediabetic conditions using human sera from non-diabetic (non-T2D) or incident diabetic (incident-T2D) in vitro models. The model based on human sera also mimics a prediabetic environment and provokes insulin resistance in adipocytes reducing the pAkt/Akt ratio, as we previously described [20]. The major advantage of using this in vitro model is the inclusion of several external prediabetic environments, that are not included in the hyperglycemia/hyperinsulinemia (HGHI model).

Cells were transfected with HA-TfR (HA-TfR) and exposed to human sera from non-diabetic (non-T2D) or incident diabetic (incident-T2D) subjects for 24 h [20]. Subsequently, cells were stimulated with holo-Tf and TfR translocation was analyzed using an anti-HA antibody.

First, we evaluated the TfR time-course internalization under holo-Tf stimulus (20, 30, and 40 min) in basal conditions. Figure 4E shows the internalization of TfR from the plasma membrane to cytosol at 20 min, into a characteristic juxta-nuclear position at 30 min. Finally, after 40 min, the juxta-nuclear position is reduced and TfR is partially recycled between the cell surface and intracellular compartments. Following, we analyzed the impact of insulin resistance on TfR internalization by exposing cells to non-diabetic or incident-diabetic conditions. Additional positive control of serum exposition using FBS was included. Functional tracking of recycling endosomes via TfR reveals perinuclear foci and cytoplasmic dots, characteristic of classical recycling, in control cells (with FBS) and non-diabetic cells (Figure 4E). In contrast, in cells exposed to incident-T2D sera, TfR is observed in sparse puncta at the periphery of cells indicating an alteration in TfR recycling (Figure 4E). In fact, incident-T2D showed a significant decrease in perinuclear localization vs. the non-T2D group (Figure 4F).

Although both conditions showed a TfR internalization, corresponding to iron uptake, incident-T2D showed internal TfR localization differences mainly related to internalization time response/recovery that could be related to the initial deleterious insulin response process observed in incident-T2D subjects.

### 3.6. Effect of Transferrin Protein Carbonylation on Molecular Interactions

**Figure 5:**
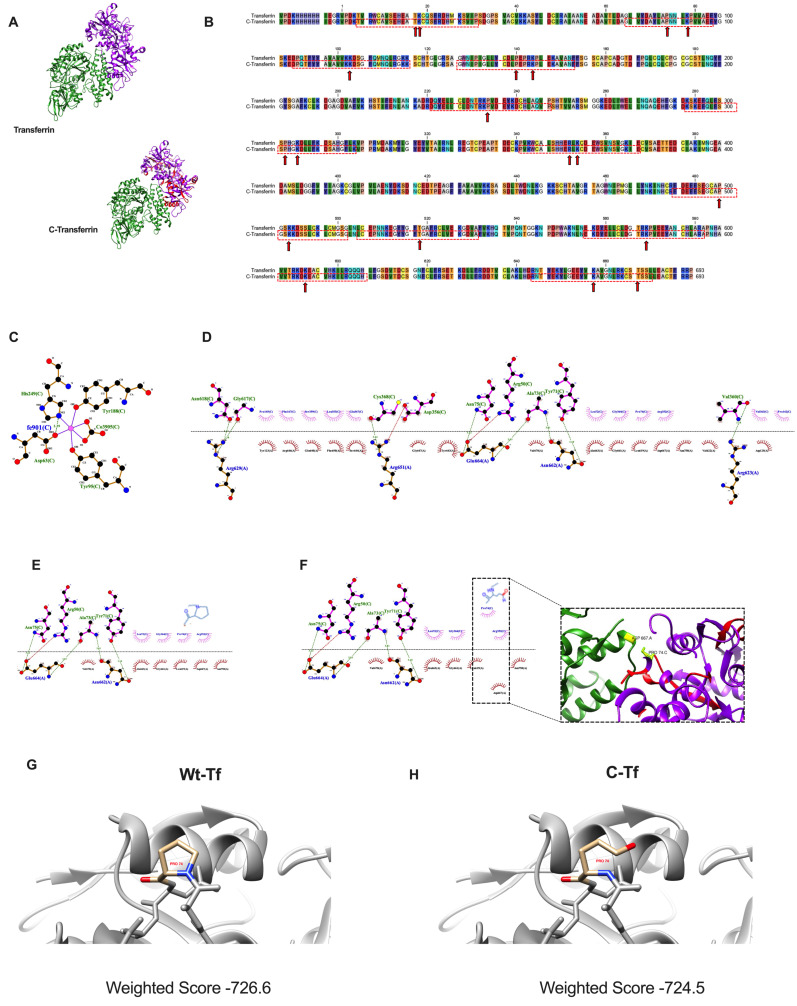
The effect of transferrin carbonylation on transferrin receptor interaction. (**A**) Tridimensional structure of transferrin (Protein Data Bank) and carbonylated transferrin (C-transferrin) showing the localization of possible carbonylation residues (in red). (**B**) Alignment of nucleotide sequence of transferrin-transferrin receptor interaction. Red boxes represent predicted carbonylated residues based on bibliography and user-friendly webserver for iCar-PseCp (**C**) 2D representation using Ligplot+ of interactions for the transferrin and iron and (**D**) transferrin and transferrin receptor. A representative chain of each protein is shown (Chain C, for TfR and chain A, for Tf). The side chains are shown in ball-and-stick representation, with the ligand bonds TfR in pink and Tf, in brown. Hydrogen bonds are shown as green dotted lines, the spoked arcs represent residues involved in hydrophobic contacts, and atoms with short lines correspond to atoms involved in hydrophobic contacts. (**E**,**F**) Fragment magnification of 2D representation of TfR-Tf interaction (**E**) and for the carbonylated transferrin (carbonylated proline) and transferrin receptor (**F**). The carbonylated proline residue in C-Tf could modify the hydrophobic contact between PRO74 of transferrin and ASP667of transferrin receptor. (**G**,**H**) Predicted interaction of TfR with Tf (**G**) or C-Tf (**H**) with corresponding weighted score, that represents the lowest energy for each cluster obtained with ClusPro.

To characterize the role of Tf carbonylation on iron homeostasis, and validate data obtained in translocation studies, we performed in silico molecular docking studies using a complex between TfR 1 and Tf with iron in the N-Lobe (PDB code: 3s9l). Both the receptor and the protein are protein dimers formed by two equal chains, so to facilitate the interaction study, equal chains (Chain B for TfR and chain D for Tf) had been removed. A representative complex between Tf (Figure 5A upper) or C-Tf (Figure 5A lower) with iron and TfR was observed. Red zones represent predicted carbonylated regions in the 3D complex. A comparative lineal sequence between Tf and C-Tf showed specific predicted carbonylated residues (red lines) indicating specific carbonylated amino acids (red arrows) on proline, lysine, arginine, and/or threonine included in the carbonylated regions (Figure 5B).

The interaction Tf-iron involves four amino acids including H249, D63, Y95, and Y188 (Figure 5C) However, no carbonylated peptides were predicted in these regions (Figure 5B) suggesting that the carbonylation of transferrin did not affect iron content. In fact, data are according to adipose tissue iron content observed in the subjects evaluated.

The interaction Tf-TfR also involves another seven specific amino acids of Tf including V360, C368, Y71, A73, N75, N618, and G617 (Figure 5D). Among them, three amino acids (V360, A73, and N75) are included in predicted carbonylated regions (Figure 5B) and are close to carbonylated amino acids, as proline (Figure 5E) compromising the interaction with TfR, via changes in hydrophobic interaction (Figure 5F). These results could explain, in part, the deleterious internalization of TfR observed in a cell exposed to prediabetic conditions.

The predicted carbonylated model of Tf (C-Tf) was similar to non-carbonylated Tf (Wt-Tf), however, differences in the weighted score were observed being lower in C-Tf vs. Wt-Tf (Figure 5G,H). These data suggest the interaction of TfR with either Wt-Tf or C-Tf uses, may be regulated by this post-translational modification, mainly driven by the hydrophobic residues.

## 4. Discussion

Carbonylation is considered the main hallmark of oxidative damage in tissues. This nonenzymatic irreversible post-translational protein modification drives protein dysfunction and degradation, affecting multiple cell signaling pathways [3,4]. Since carbonylation is an irreversible process, it would be interesting to identify pathways affected by protein carbonylation to elucidate alternative pathways that balance protein carbonylation dysfunction.

First, we analyzed possible oxidative stress markers at systemic levels. Classical oxidative stress biomarkers (PC, LPO, TG, or GPx) were measured in obese subjects with different insulin sensitivity degrees (Ob-NG, Ob-IR, and Ob-T2D); however, no differences were observed between groups. In fact, no significant differences in plasma parameters were observed in these subjects, except in glucose homeostasis according to the classification of the subjects. However, positive correlations were detected between glucose parameters and plasmatic ferritin and iron (Supplementary Figure S1) showing a possible role of iron homeostasis in insulin resistance in these subjects. Along this line, several studies evidence the association between iron overload and insulin resistance [24,25,26,27,28]. However, the molecular mechanism involved has not yet been clarified. Circulating iron is bound to Tf and entered into the cell via TfR and insulin is able to stimulate iron uptake by the cells, but the interaction between iron and insulin is modulated, in part by oxidative stress [28]. Taking into account that oxidative stress and redox status are affected by obesity and metabolic risk in adipose tissue [29], we evaluate redox status in our subjects in both, OM and SC fat depots.

Data obtained from the OM fat depot showed an increase in ROS production in Ob-IR and Ob-T2D, which could induce a high risk of protein carbonylation. In contrast, the SC fat depot did not show differences in ROS status. However, correlations between plasma iron homeostasis parameters and oxidative stress markers are detected in both depots (ferritin vs. DNP, r = 0.669, *p* = 0.049 in SC fat depot) (Tf vs. intracellular ROS, r = 0.62, *p* = 0.031 in OM fat depot). Data highlight a relationship between iron homeostasis and adipose tissue oxidative damage in obesity-associated insulin resistance. These results are according to previous research, i.e., OM is more sensitive to oxidative stress dysfunction than SC fat depot [29]. However, no molecular changes associated with these cause-and-effect relationships were described.

In our study, we describe for the first time the OM and SC adipose tissue carbonylome in obese subjects with different insulin sensitivity. Thus, the main carbonylated proteins affected in adipose tissue in our study including insulin resistance associated with obesity are related to ER stress, cytoskeletal reorganization, and metabolism. Similar carbonylated proteins related to these processes, especially metabolism and structure process, were previously described in adipose tissue in relation to increasing body weight in the SC human adipose tissue and animal model [10]. However, no changes associated with insulin resistance, independent of body weight, have been described yet. Along this line, the most significant changes were detected in the OM fat depot increasing carbonylated protein expression of serpin A1, Tf, and vimentin in Ob-IR subjects. These proteins are closely related to metabolic dysfunction and could explain part of the deleterious effect observed in obese adipose tissue [26,30,31,32,33,34]. Thus, proteomic data evidence depot-specific differences in adipose tissue carbonylome and provide novel insights into the association of OM adipose tissue and obesity-associated insulin resistance.

Additional studies showed a correlation between adipose oxidative stress and iron metabolism and evidence of a dysregulation of iron homeostasis in T2D [24,25,28], the molecular mechanism involved in this correlation has not been elucidated yet. Thus, we focused on iron uptake via TfR, due to the relevance of TfR translocation into GLUT4 transport [35,36,37]. Thus, GLUT4 is sorted from TfR into a specialized perinuclear reticular GLUT4 storage compartment [36]. Our studies on TfR internalization evidence a decrease in the perinuclear localization of TfR in adipocytes exposed to sera from incident T2D subjects compared to non-T2D subjects, which could be explained, by a potential alteration in glucose homeostasis in these incident-T2D subjects.

Moreover, molecular docking studies revealed specific carbonylated regions that could compromise the functionality of transferrin, and the interaction with TfR, partially affecting glucose uptake. In our study, we use a complex including TfR, Tf, and iron due to iron release in the endosome occurring without dissociation of Tf from TfR [38]. Tf is an ~80 kDa bilobal (N- and C-lobes) glycoprotein that binds iron from plasma into the cell via TfR interaction. In turn, TfR is a dimeric transmembrane protein, with a small cytoplasmic domain, a single-pass transmembrane region, and a large extracellular domain. Each monomer has three structurally distinct domains: a protease-like domain proximal to the membrane, a helical domain accounting for all the dimer contacts, and a membrane-distal apical domain. Interactions of C and N-lobe of Tf with TfR have been described, nevertheless, interactions of the N-lobe with TfR are probably more complex [39] and we used these to describe the role of Tf carbonylation on interaction with TfR. The interaction between the N2 domain of Tf includes two prolines, two basic residues, and possibly an acidic residue. This arrangement suggests the binding site is composed of hydrophobic and ionic interactions [39]. In fact, our predictive model of C-Tf identifies one carbonylated proline (PRO74), localized in a specific region of Tf-TfR interaction, that modulates hydrophobic interaction with ASP667, affecting the ligand-receptor complex. Additionally, a docking simulation using carbonylated-Tf was performed using ClusPro. Compared with WT-Tf, the interaction TfR-Tf carbonylated revealed a lower weighted score showing changes in the interaction interface. These results are interesting for the understanding of protein–protein interactions (PPIs) having pivotal roles in life processes [40,41]. In fact, there is an increasing interest in targeting PPIs as a novel strategy for the development of new drugs [41].

The main limitation of the study is based on molecular tools to validate specific carbonylated proteins. Nonetheless, our study reveals a significant increment of C-Tf expression in Ob-IR subjects, a deleterious transferrin receptor recycling in adipocytes exposed to prediabetic conditions, and lower binding between the interaction C-Tf- TfR, evidencing the possible role of carbonylated transferrin in insulin resistance in adipocytes. Additional data related to C-Tf, both human and in vitro models of adipocytes, will be necessary to unravel the role of C-Tf in adipose tissue dysfunction related to insulin resistance conditions.

## 5. Conclusions

In conclusion, our proteomic data revealed depot-differences in adipose tissue carbonylome from obese-associated insulin resistance, highlighting the interaction between iron-glucose transport and redox status in obesity-associated insulin resistance.

## Figures and Tables

**Table 1:** Anthropometric and biochemical characteristics of subjects.

	Ob-NG (n = 20)	Ob-IR (n = 37)	Ob-T2D (n = 16)	ANOVA
Sex (men/women)	8/12	11/26	8/8	
Age (years)	34.50 ± 2.26	42.14 ± 1.68 ^a^	44.69 ± 2.02 ^aa^	<0.01
Lipid-lowering therapy (n, %)	2, 10	7, 19	4, 25	
Antidiabetic therapy (n, %)	2, 10	2, 5.4	8, 50	
Antihypertensive therapy (n, %)	3, 15	18, 48.6	7, 43.8	
Weight (kg)	143.90 ± 6.5	148.60 ± 6.14	156.4 ± 6.26	ns
BMI (kg/m^2^)	52.38 ± 2.37	53.25 ± 1.80	54.53 ± 1.49	ns
Waist circumference (cm)	136.6 ± 4.06	145.3 ± 3.85	149.0 ± 3.53	ns
Glucose (mg/dL)	90.95 ± 2.00	107.9 ± 1.66 ^aaa^	171.4 ± 9.92 ^aaa,bbb^	<0.0001
HbAc1 (mmol/mol)	5.64 ± 0.12	6.24 ± 0.22 ^aa^	9.17 ± 0.5 ^aaa,bbb^	<0.0001
HOMA-IR	3.31 ± 0.37	5.49 ± 0.44 ^aa^	8.44 ± 1.42 ^aaa^	<0.001
Insulin (mU/L)	14.72 ± 1.5	21.06 ± 1.73	19.88 ± 2.56	ns
Triglycerides (mg/dL)	121.1 ± 12.42	117 ± 7.91	195 ± 30.71 ^aa,bb^	<0.001
Cholesterol (mg/dL)	182 ± 10.68	201.1 ± 5.96	195.8 ± 9.5	ns
HDL (mg/dL)	40.47 ± 3.05	41.86 ± 1.5	35.5 ± 1.66	ns
LDL (mg/dL)	121.2 ± 8.47	136 ± 4.9	118.6 ± 7.99	ns
Hemoglobin (g/dL)	13.74 ± 0.38	13.31 ± 0.28	14.08 ± 0.47	ns
Hematocrit (%)	41.52 ± 0.94	39.47 ± 1.25	42.61 ± 1.11	ns
Iron (ug/dL)	82.42 ± 6.28	68.35 ± 5.33	69.13 ± 7.74	ns
Ferritin (ng/mL)	83.59 ± 18.04	98.54 ± 15.09	149.9 ± 27.56	ns
Transferrin (mg/dL)	254 ± 12.39	279.6 ± 8.04	276.1 ± 10.07	ns
PC (pM/L)	8.22 ± 0.88	8.54 ± 1.01	8.49 ± 1.01	ns
LPO (μM/L)	5.75 ± 0.76	5.74 ± 1.00	5.47 ± 0.64	ns
NOx (μM/L)	29.5 ± 1.18	28.8 ± 1.67	42.8 ± 2.84	ns
GSH (mmol/mg Hb)	1.5 × 10^−5^ ± 1.9 × 10^−6^	1.6 × 10^−5^ ± 5.9 × 10^−6^	1.2E-5 ± 1.0 × 10^−6^	ns
GT (mmol/mg Hb)	1.8 × 10^−5^ ± 2.2 × 10^−6^	2.1 × 10^−5^ ± 9.1 × 10^−6^	1.4E-5 ± 1.1 × 10^−6^	ns
GPx (UI/mg prot)	2.44 ± 0.06	2.33 ± 0.03	2.28 ± 0.02	ns

BMI, body mass index; HbAc1, glycated hemoglobin; HOMA-IR, homeostasis model assessment of insulin resistance; HDL, high-density lipoprotein; LDL, low-density lipoprotein; PC, protein carbonyls; LPO, lipid peroxidation products; NOx, total nitrites; GSH, reduced glutathione; GT, glutathione total; GPx, glutathione peroxidase. Data represent the mean ± SEM. p-value was analyzed using one-way analysis of variance (ANOVA) and corresponding post-hoc test. ^a^ *p* < 0.05, ^aa^ *p* < 0.01, ^aaa^ *p* < 0.001 vs. NG obese subjects; ^bb^ *p* < 0.01, ^bbb^ *p* < 0.001 vs. IR obese subjects; ns, not significant.

**Table 2:** Carbonylated proteins identified by MALDI-TOF/TOF in human adipose tissue.

Spot Num. ^a^	Protein Name	Symbol	Accession Number ^b^	MW (KDa)/pI	%Cover. ^c^	Pep. ^d^	Score
Omental adipose tissue (OM depot)							
1	Transferrin	TRFE_HUMAN	P02787	79.3/6.81	24	19	222
2	Transferrin	TRFE_HUMAN	P02787	79.3/6.81	35	32	613
3	Transferrin	TRFE_HUMAN	P02787	79.3/6.81	39	35	695
4	Serum albumin	ALBU_HUMAN	P02768	71.3/5.92	54	38	760
5	Alpha-1-antitrypsin	A1AT_HUMAN	P01009	46.9/5.37	43	19	297
6	Alpha-1-antitrypsin	A1AT_HUMAN	P01009	46.9/5.37	25	14	106
7	Alpha-1-antitrypsin	A1AT_HUMAN	P01009	46.9/5.37	43	19	252
8	Vimentin	VIME_HUMAN	P08670	53.7/5.05	61	32	512
9	Vimentin	VIME_HUMAN	P08670	53.7/5.05	54	30	548
10	Tubulin beta-4B chain	TBB4B_HUMAN	P68371	50.3/4.79	27	14	157
11	Alpha-1-antitrypsin	A1AT_HUMAN	P01009	46.9/5.37	40	18	368
12	Vimentin	VIME_HUMAN	P08670	53.7/5.05	51	25	454
13	Vimentin	VIME_HUMAN	P08670	53.7/5.05	59	34	548
14	Vimentin	VIME_HUMAN	P08670	53.7/5.05	31	23	378
15	Vimentin	VIME_HUMAN	P08670	53.7/5.05	42	26	403
16	Vimentin	VIME_HUMAN	P08670	53.7/5.05	31	19	241
17	Not detected						
18	Not detected						
19	Actin, cytoplasmic 1	ACTB_HUMAN	P60709	42.1/5.29	27	18	352
20	Actin, cytoplasmic 1	ACTB_HUMAN	P60709	42.1/5.29	57	21	488
21	Vimentin	VIME_HUMAN	P08670	53.7/5.05	47	30	452
22	Actin, cytoplasmic 1	ACTB_HUMAN	P60709	42.1/5.29	53	26	487
23	Annexin A2	ANXA2_HUMAN	P07355	38.8/7.57	52	22	430
Subcutaneous adipose tissue (SC depot)							
1	Transferrin	TRFE_HUMAN	P02787	79.3/6.81	21	15	198
2	Transferrin	TRFE_HUMAN	P02787	79.3/6.81	27	21	259
3	Not detected						
4	Serum albumin	ALBU_HUMAN	P02768	71.3/5.92	58	40	686
5	Vimentin	VIME_HUMAN	P08670	53.7/5.05	56	30	510
6	Vimentin	VIME_HUMAN	P08670	53.7/5.05	28	14	147
7	Vimentin	VIME_HUMAN	P08670	53.7/5.05	17	9	43
8	Vimentin	VIME_HUMAN	P08670	53.7/5.05	42	22	432
9	Vimentin	VIME_HUMAN	P08670	53.7/5.05	23	11	123
10	Not detected						
11	Not detected						
12	Not detected						
13	Vimentin	VIME_HUMAN	P08670	53.7/5.05	29	15	188
14	Actin, cytoplasmic 2	ACTG_HUMAN	P60709	42.1/5.29	46	15	379
15	Not detected						
16	Not detected						
17	Annexin A2	ANXA2_HUMAN	P07355	38.8/7.57	50	24	553
18	Actin, cytoplasmic 2	ACTG_HUMAN	P60709	42.1/5.29	30	9	159
19	Carbonic anhydrase 1	CAH1_HUMAN	P00915	28.9/6.59	57	15	401
20	Not detected						
21	Alpha-crystallin B chain	CRYAB_HUMAN	P02511	20.1/6.76	48	8	59
22	Alpha-crystallin B chain	CRYAB_HUMAN	P02511	20.1/6.76	57	14	237

^a^ Spot numbers correspond to those in Figure 2A. ^b^ Accession number from the NCBInr database. ^c^ Coverage of all peptide sequences matched to the identified protein sequence (%). ^d^ Pep. corresponds to the number of peptides identified (Mascot).

## Data Availability

Not Applicable.

## Supplementary Materials

The following supporting information can be downloaded at: https://www.mdpi.com/article/10.3390/biomedicines10123032/s1, Figure S1: Correlation studies of plasma parameters in obesity and insulin resistance, Figure S2: Workflow and validation of carbonylome in insulin-resistance in vitro model in 3T3-L1 adipocytes. Table S1. Antibodies.
