# Supplementary material for: Influence of Protein Carbonylation on Human Adipose Tissue Dysfunction in Obesity and Insulin Resistance
Source: Biomedicines. 2022 Nov 24;10(12):3032. doi: 10.3390/biomedicines10123032 (PMC9775537; doi:10.3390/biomedicines10123032)
Supplement: Supplementary file 1 [file biomedicines-10-03032-s001.zip › Supplementary Figures.pdf]

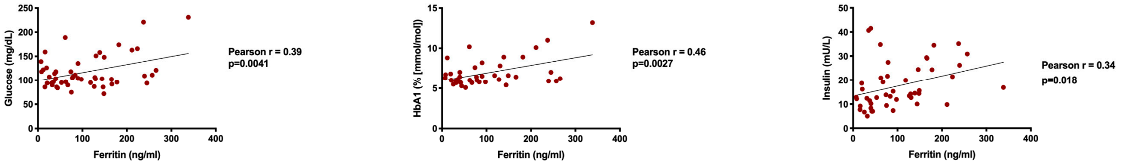

Supplementary Figure 1. Correlation studies of plasma parameters in obesity and insulin resistance. r, Pearson's correlation coefficient from iron homeostasis markers and plasma parameters in obese subjects (Ob-NG, Ob-IR and Ob-T2D). *p<0.05, **p<0.01 and ***p<0.001

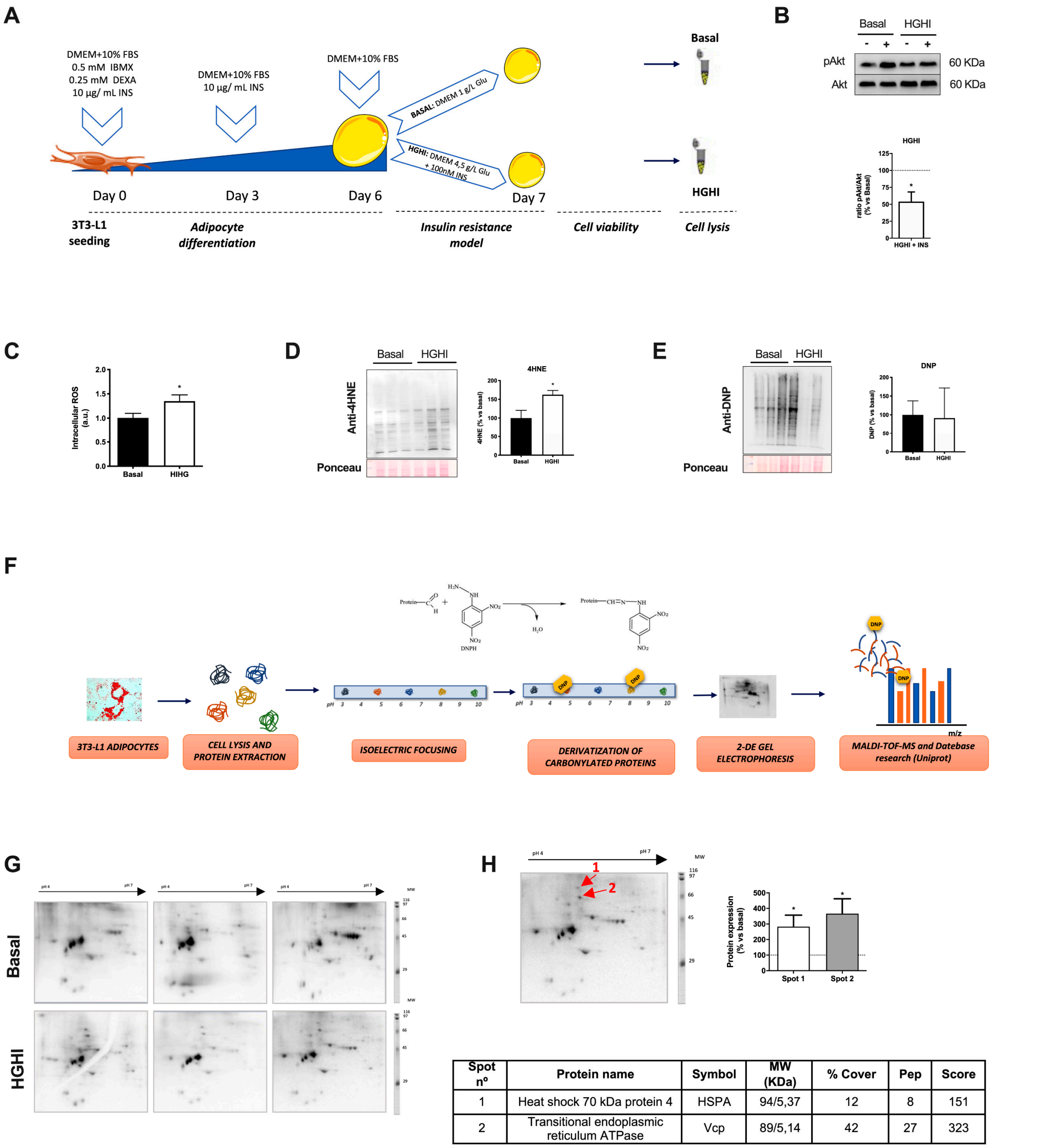

Spot nº	Protein name	Symbol	MW (KDa)	% Cover	Pep	Score
1	Heat shock 70 kDa protein 4	HSPA	94/5,37	12	8	151
2	Transitional endoplasmic reticulum ATPase	Vcp	89/5,14	42	27	323

Supplementary Figure 2. Workflow and validation of carbonylome in insulin-resistance *in vitro* model in 3T3-L1 adipocytes. (A) General workflow for the cell culture and *in vitro* model in 3T3-L1 adipocytes (D6) in basal condition (Low Glucose 1g/L) or under high glucose (4,5g/L) and high insulin (100nM) stimulus (HGHI) during 24h. **(B)** Insulin response (pAkt/Akt ratio) of 3T3-

L1 cells exposed to HGHI. After the treatment, basal and HGHI-treated 3T3-L1 cells were stimulated with insulin (100 nM) for 5 min. Cell lysates were analyzed for Akt and pAkt content. **(C)** Intracellular ROS in basal and HGHI-treated 3T3-L1 cells. **(D,E)** Quantification of carbonylated proteins using 4-HNE-modified proteins (D) and DNP-protein adducts (E) in basal and HGHI-treated 3T3-L1 cells. Immunoblot images (left panel) and quantification (right panel) are shown. For quantification, protein levels were normalized using Ponceau staining. **(F)** General workflow for the carbonylome analysis in 3T3-L1 adipocytes in basal or exposed to HGHI conditions. **(G)** Two-dimension gel electrophoresis of total carbonylated protein in basal and HGHI-treated 3T3-L1 cells using anti-DNP antibody. **(H)** Representative image of total 3T3-L1 proteome of pooled extract from basal and HGHI-treated 3T3-L1 cells using Coomassie staining (left panel). Proteins were separated on a 2-DE gel using 4 cm pH 4–7 NL strips in the first dimension and 10% SDS-PAGE gels in the second dimension. Molecular weight standards were loaded on the second dimension. Differentially expressed proteins between conditions are indicated with arrows. Quantification of differentially expressed carbonylated proteins (spots) between groups (right panel). The numbers correspond to the spot numbers are listed in bottom table. Data are expressed as mean±SEM, as percentage *vs* basal stimulus (100%) of four independent experiments. *$p<0.05$, *vs* basal using unpaired T test.
