# Supplementary material for: Influence of Protein Carbonylation on Human Adipose Tissue Dysfunction in Obesity and Insulin Resistance
Source: Biomedicines. 2022 Nov 24;10(12):3032. doi: 10.3390/biomedicines10123032 (PMC9775537; doi:10.3390/biomedicines10123032)
Supplement: Supplementary file 1 [file biomedicines-10-03032-s001.zip › Supplementary Table.pdf]

Supplementary Table 1. List of Antibodies.

**Antibody**	**Host specie**	**Reference**	**Commercial Source**	**Dilution**
Anti-Dinitrophenyl-KLH (DNP)	Rabbit	A64535	RD Systems	1:2500
Anti-4HNE	Mouse	MAB3249	*RD system*	1:2000
Anti-GSS	Rabbit	ab290	*Abcam*	1:1000
Anti-SOD1	Rabbit	ab45422	*Abcam*	1:1000
Anti-HA	Goat	Sc-16147	*Santa Cruz*	1:1000
Anti-Rabbit IgG peroxidase	Rabbit	A8275	*Sigma Aldrich*	1:2500
Anti-Mouse IgG peroxidase	Mouse	A-9044	*Sigma Aldrich*	1:2500
Anti-Goat/Sheep IgG peroxidase	Goat	A-9452	*Sigma Aldrich*	1:2500
