# Supplementary figures and images for: Influence of Protein Carbonylation on Human Adipose Tissue Dysfunction in Obesity and Insulin Resistance
Source: Biomedicines. 2022 Nov 24;10(12):3032. doi: 10.3390/biomedicines10123032 (PMC9775537; doi:10.3390/biomedicines10123032)

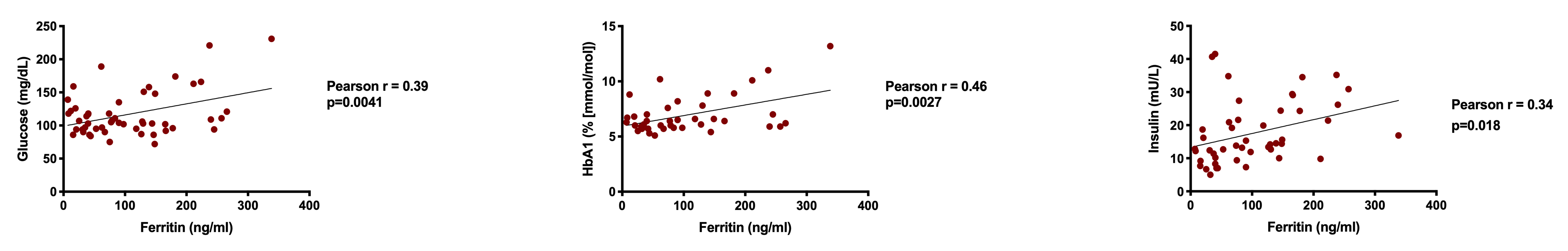

Supplement: Supplementary file 1 [file biomedicines-10-03032-s001.zip › Figure S1.tiff]

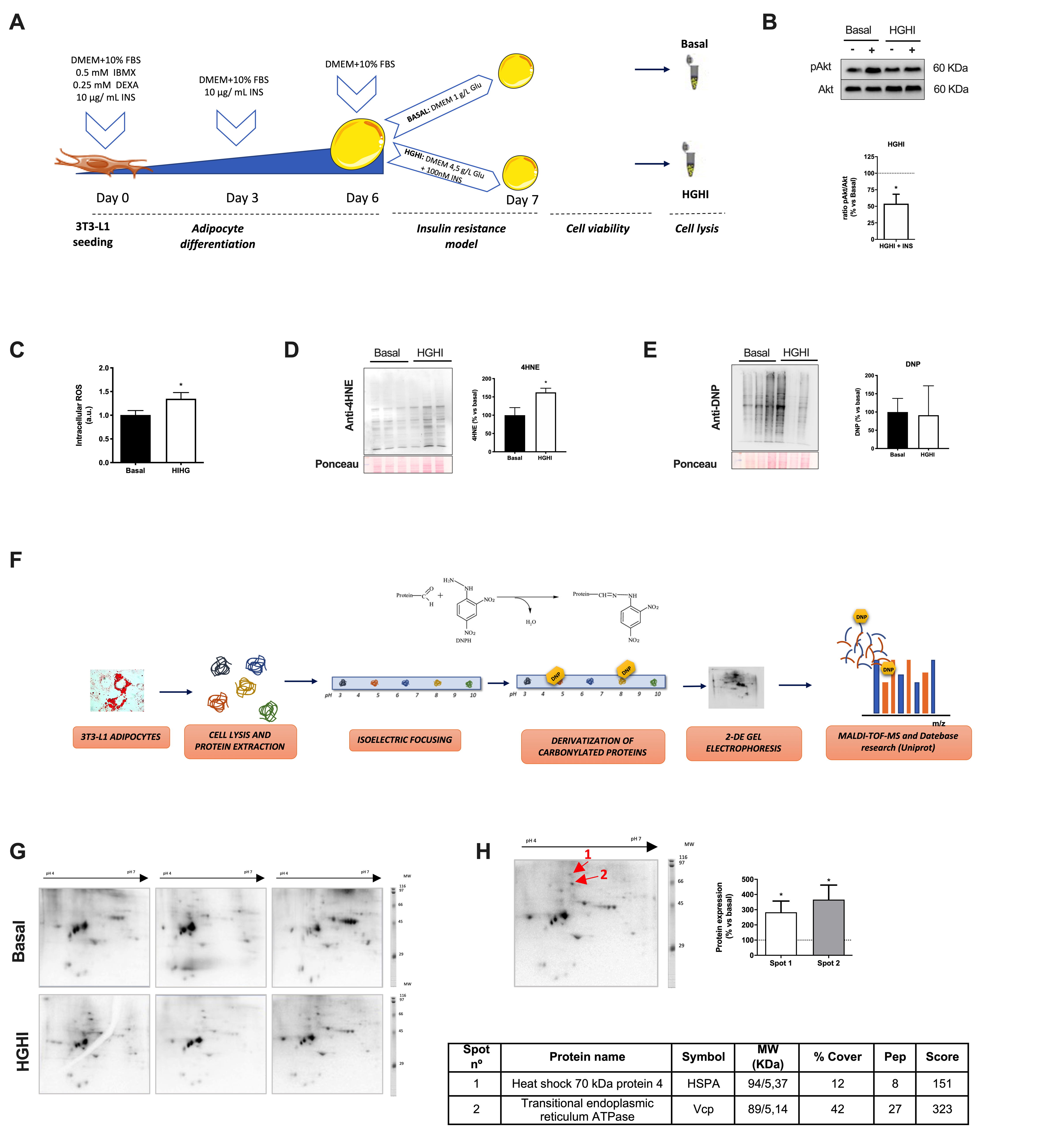

Supplement: Supplementary file 1 [file biomedicines-10-03032-s001.zip › Figure S2.tiff]
